# Biotransformation of chromium by root nodule bacteria *Sinorhizobium* sp. SAR1

**DOI:** 10.1371/journal.pone.0219387

**Published:** 2019-07-30

**Authors:** Renitta Jobby, Pamela Jha, Anand Gupta, Arpita Gupte, Neetin Desai

**Affiliations:** 1 Amity Institute of Biotechnology, Amity University, Mumbai-Pune Expressway, Bhatan, Post–Somathne Panvel, Mumbai, India; 2 School of Biotechnology and Bioinformatics, D. Y. Patil Deemed to be University, Sector15/50, CBD, Belapur, Navi Mumbai, India; Babasaheb Bhimrao Ambedkar University, INDIA

## Abstract

The present study aims to address the problem of chromium (Cr) toxicity by providing important insights into the mechanisms involved in its bioremediation. Among the 22 *Rhizobium* and *Sinorhizobium* isolates obtained from *Sesbania sesban* root nodules, *Sinorhizobium* sp. SAR1 (JX174035.1) tolerated the maximum Cr concentration (1mM) and hence was used for further studies. The excess secretion of extra polymeric substances, as seen from scanning electron micrographs, could be a probable mechanism of adaptation to the Cr stress. The Energy dispersive X-ray spectroscopy data did not show any peaks of Cr. The biosorption studies done on the isolate gave maximum adsorption capacity as 285.71mg/g. The isotherm studies showed a better fit to Langmuir isotherm. The Weber and Morris plot established that the phenomenon of adsorption was governed by film diffusion mechanism. The FTIR analysis suggested the role of cell wall components and extracellular polymeric substances in Cr adsorption to the biomass of *Sinorhizobium*. On the basis of these results a compiled mechanism of Cr (VI) adsorption and its biotransformation into Cr (III) by *Sinorhizobium* sp. SAR1 is explained. This work outlines a comprehensive detail for the exact phenomenon of Cr biotransformation by *Sinorhizobium* sp. SAR1. These results may further help in developing and enhancing effective bioremediation approaches.

## Introduction

Human activities have proven to immensely compromise the harmony that existed between him and his environment. Metals are useful in all walks of human life. Unfortunately, the past century has witnessed concerning levels of heavy metal contamination. Among the toxic heavy metals, chromium (Cr) is being focused, since it finds a wide range of application in the industrial production of steel, metal alloys, cement, galvanized plastic, leather, paints, fertilizers and fungicides [1]. Tanneries are one of the major contributors to excessive Cr in the environment. They generate around 40 million tons of Cr contaminated wastes [2]. Studies have revealed severe contamination of productive agricultural land and water bodies by tannery waste disposal in developing (i.e. India, Bangladesh) as well as developed (i.e. Australia) countries [3, 4].

The most stable forms of Cr i.e., the hexavalent and trivalent Cr are found in abundance, in nature, as compared to its other oxidation states [5, 6]. Among these, the hexavalent form of Cr is a proven carcinogen and mutagen. It is also considered to be more toxic than the trivalent form [7, 8].

The conventional methods for Cr (VI) removal generate a large volume of sludge which again creates secondary pollution problems. The growing environmental awareness has emphasized the development of environment-friendly ways of decontamination procedures [7, 9]. In this regard, the biological treatment protocols have evoked great interest among researchers. Bioremediation can effectively substitute the conventional cleanup technologies [10]. It refers to the process of propagating living systems which are able to adsorb and/or metabolize pollutants, thus allowing detoxification. Bioremediation of heavy metals from aqueous solutions is possible with the help of micro-organism, due to their characteristic properties of bioaccumulation, biosorption and enzymatic reduction [11, 12]. Bacteria generally detoxify chromium by reduction of Cr (VI) to less toxic Cr (III) [13]. Presently, the scope of various Plant-Growth Promoting Rhizobacteria (PGPR) is screened for its ability of metal removal. These bacteria may prove extremely valuable in the treatment of metal contaminated soils. The PGPR not only shows potential as a biofertilizer but also increases the tolerance to heavy metal induced stress in plants [14, 15]. Rhizobia are considered to be a well-known group of PGPR with multipotential property to be used as a biofertilizer as well as in bioremediation [7, 16].

The main objective of the present study was to isolate a Cr resistant *Rhizobium* sp., study the mechanisms involved in Cr resistance, and check the efficiency of the isolates in Cr bioremediation.

## Materials and methods

### Bacterial culture

Twenty-two isolates of *Rhizobium* and *Sinorhizobium* obtained from root nodules of *Sesbania sesban* from industrial areas of Navi Mumbai were used for this study [17].

### Metal resistance studies

Analytical grade of Potassium dichromate (K_2_Cr_2_O_7_) obtained from Molychem was used to prepare stock solutions of the metal. The stock solution was filter sterilized and added to Tryptone Yeast extract (TY) agar to obtain a final concentration of (0.01-10mM) of Cr for determination of MIC. The cultures were grown in TY broth until log phase was reached and 50μL was spot inoculated onto the TY plates prepared with different concentrations of metals. It was incubated at 25°C ± 2°C and observed for growth every alternate day over the entire week. The TY plates without metal were used as control. The percentage susceptibility towards Cr was calculated as:

$$\frac{Total\;no.of\;isolates-no.of\;isolates\;that\;did\;not\;grow}{Total\;no.of\;isolates}x100$$

The MIC was recorded as the lowest concentration of Cr that prevented the growth of isolates on TY plates. All the experiments were carried out in triplicates. Log phase cultures showing resistance on TY plates were further inoculated in TY broth, supplemented with suitable concentrations of Cr (VI) to determine its MIC for test isolates. The broths were incubated under shaking condition (120rpm) at 25°C ± 2°C. The absorbance was measured at 600nm for one week. The isolates showing highest metal tolerance were selected for further studies.

### Scanning electron microscopy (SEM) and Energy dispersive X-ray spectroscopic (EDS) analysis

The Cr (VI) resistant isolates obtained in our study were subjected to SEM and EDS analysis. For this purpose, the isolates were grown in TY broth with (sample) and without (control) Cr (VI) until they reached a log phase. The broths were incubated under shaking condition (120rpm) at 25°C (± 2°C). The log phase cultures (1.5mL), were centrifuged at 10,000rpm for 10min and the pellet thus obtained was washed twice with phosphate buffered saline (1X). It was then fixed using 2.5% glutaraldehyde prepared in double distilled water (DDW) for 2h. The fixed cells were washed twice with DDW, dehydrated with a series of ethanol concentration from 25% to 100% for 5min each, and subsequently left to dry overnight in a desiccator [18]. The specimens were mounted onto the sample holder with carbon-conductive adhesive tapes and coated with gold using a sputter coater (Auto fine coater JFC-1600, JEOL) prior to viewing, using a field-emission scanning electron microscope (FESEM JSM-7600F, JEOL) fitted with EDS analyzer.

### Chromium removal studies

#### Preparation of the bacterial biosorbent

The isolate was pre-cultured in TY medium till early-stationary phase and the biomass obtained was used for biosorption studies. A weighed amount of wet biomass was oven dried at 80°C until it exhibited a constant weight. The final weight determined was used to calculate its dry weight.

#### Optimization of parameters for chromium removal

The effect of parameters like pH (1–9), temperature (25°C-45°C) and biomass dosage (1.25g/L-20g/L) was studied on the removal of Cr (VI) by test isolates. The stock solution (1000mg/L) of Cr (VI) was prepared using K_2_Cr_2_O_7_ (Molychem, Mumbai, Maharashtra). The initial experiments of of pH and temperature were done starting with 10mg/L of Cr (VI) solution and 2.5g/L of biomass. While later for biomass dosage studies to avoid limitation of Cr availability a Cr concentration of 100mg/L of the same was used since the highest biomass studied was 20g/L. After inoculation of the biomass into the Cr (VI) solutions, the flasks were kept at 28°C under shaking condition (120rpm). The aliquots were taken after 10min, 24h, 48h, 72h, 96h and 120h, and centrifuged at 10,000rpm for 10mins to remove biomass. Controls without biomass were also maintained for all the studies.

#### Analytical techniques for chromium estimation

The Diphenylcarbazide (DPC) method of Cr (VI) removal was carried out by estimating a decrease in Cr (VI) concentration in the supernatant [19]. The purple colour complex formed on reacting Cr (VI) with 1, 5- diphenyl carbohydrazide was estimated using a UV spectrophotometer (Shimadzu UV-1800, Japan). The absorbance of the same was measured at 540nm. Additionally, the total Cr ions in the supernatant were estimated using Inductively Coupled Plasma—Atomic Emission Spectrometer (ICP-AES, ACROS, M/s. Spectro, Germany). The Cr (III) content in the liquid solution was calculated as the difference between the total Cr and the content of Cr (VI). The %Cr removal was calculated as:

$$\%Cr\;removal=\frac{Initial-Final\;metal\;concentration}{Initial\;metal\;concentration}x100$$

All the experiments were carried out in triplicates and the observations were recorded as mean ± standard deviation.

#### Isotherm and kinetic studies for Cr removal

The Cr (VI) removal assays were studied using 100mg/L to 500mg/L concentration of Cr (VI). For carrying out these assays, an optimized condition of pH, temperature and biomass dosage were used. After inoculation of the biomass into the Cr (VI) solutions, the flasks were kept at 28°C under shaking condition (120rpm). The aliquots were taken from the solutions at 10min, 24h, 48h, 72h, 96h and 120h, and centrifuged at 10,000rpm for 10min. The concentration of Cr (VI) and total Cr ions in the supernatant was measured similarly as described above.

The biosorption capacity (q), in terms of mg metal/g dry cell, was calculated using the formula:

$$q=\frac{C_{i‐}C_{\mathrm{eq}}}{X}$$

Where C_i_ is the initial metal concentration (mg/L), C_eq_ is the residual metal concentration at equilibrium (mg/L) and X is the biomass concentration (g dry cell/L).

The equilibrium distribution of Cr ions between the liquid phase and solid phase of bacterial biomass was analysed by two parameter models viz., Langmuir and Freundlich isotherms. These parameters are widely used to analyse metal adsorption data. It establishes the maximum capacity of adsorption of metals (Cr VI) on adsorbents (bacterial surface) and is expressed in terms of quantity of metal adsorbed per unit mass of adsorbent used (mg/g) [20, 21]. As per the Langmuir model, the monolayer adsorption of solute occurs at a fixed number of homogeneously distributed sites over the sorbent surface. All these sites have an equal affinity for the adsorbate.

The Langmuir equation is expressed as as:

$$q=\frac{q_{\max}bC_{\mathrm{eq}}}{1+bC_{\mathrm{eq}}}$$

Where ‘q_max_’ is the maximum metal uptake under the given conditions, ‘Ceq’ is the residual metal concentration at equilibrium (mg/L), ‘b’ is a constant related to the affinity between the sorbent and sorbate. The constants in Langmuir isotherm can be determined by plotting (1/q) versus (1/C_eq_).

As per the Freundlich isotherm model, the sorption process occurs in a multilayer manner on a heterogenous surface, where sorption sites have a varied affinity for the adsorbate. The Freundlich equation isotherms is expressed as:

$$q=KC_{\mathrm{eq}}{}^{1/n}$$

Where k and n are Freundlich’s constant. The constants in Freundlich isotherm can be determined by plotting (log q) vs (log C_eq_).

To understand the kinetics of solute biosorption, a Weber and Morris Plot of q v/s √t and logq v/s 0.5log t was also plotted. This is important so as to run an effective batch biosorption system and to understand the mechanism of biosorption and the rate controlling steps. As per this model, if the rate-limiting step is intra-particle diffusion, a plot of solute sorbed against the square root of contact time should yield a straight line passing through the origin [22].

### FTIR analysis

For FTIR analysis, the biomass (7.5g/L) was subjected to 100mg/L of the metal solution and the flask was kept under shaking condition (120rpm) for 120h. Then, the pellet was dried at 60°C for 18h. It was further powdered and mixed with KBr powder for making pellet. The pellet was used for FTIR analysis (Bruker, Germany) within the range 450–4000 cm^-1^.

### Data analysis

All the experiments were setup in triplicates. The readings were statistically analyzed and presented as means with the appropriate standard deviation obtained from triplicate values.

## Result and discussion

### Screening for chromium resistance

In the present study, 22 *Rhizobium* and *Sinorhizobium* isolates were screened for their ability to tolerate hexavalent Cr. It was observed that the ability to tolerate Cr decreased with increasing metal concentration. The isolates were defined as resistant if they were able to grow in the presence of 1mM of Cr [23]. Based on this metal resistance limit, it was observed that four isolates showed resistance upto 1mM Cr concentration (S1 Table). The MIC studies in broth were also carried out for the cultures showing resistance to Cr. It was observed that the plate MIC’s were higher than those of the broth, wherein out of the four isolates, only SAR1 showed growth on 1mM of Cr in broth (S1 Fig). Hence, it was used for further analysis. The MIC values of 1mM obtained, in the present study, for the *Sinorhizobium* sp. SAR1 were higher than those obtained by Abou-Shanab et al., where *Sinorhizobium fredii* AY509217 gave MIC value of 0.1 for Cr [24].

### SEM and EDS analysis

**Fig 1:**
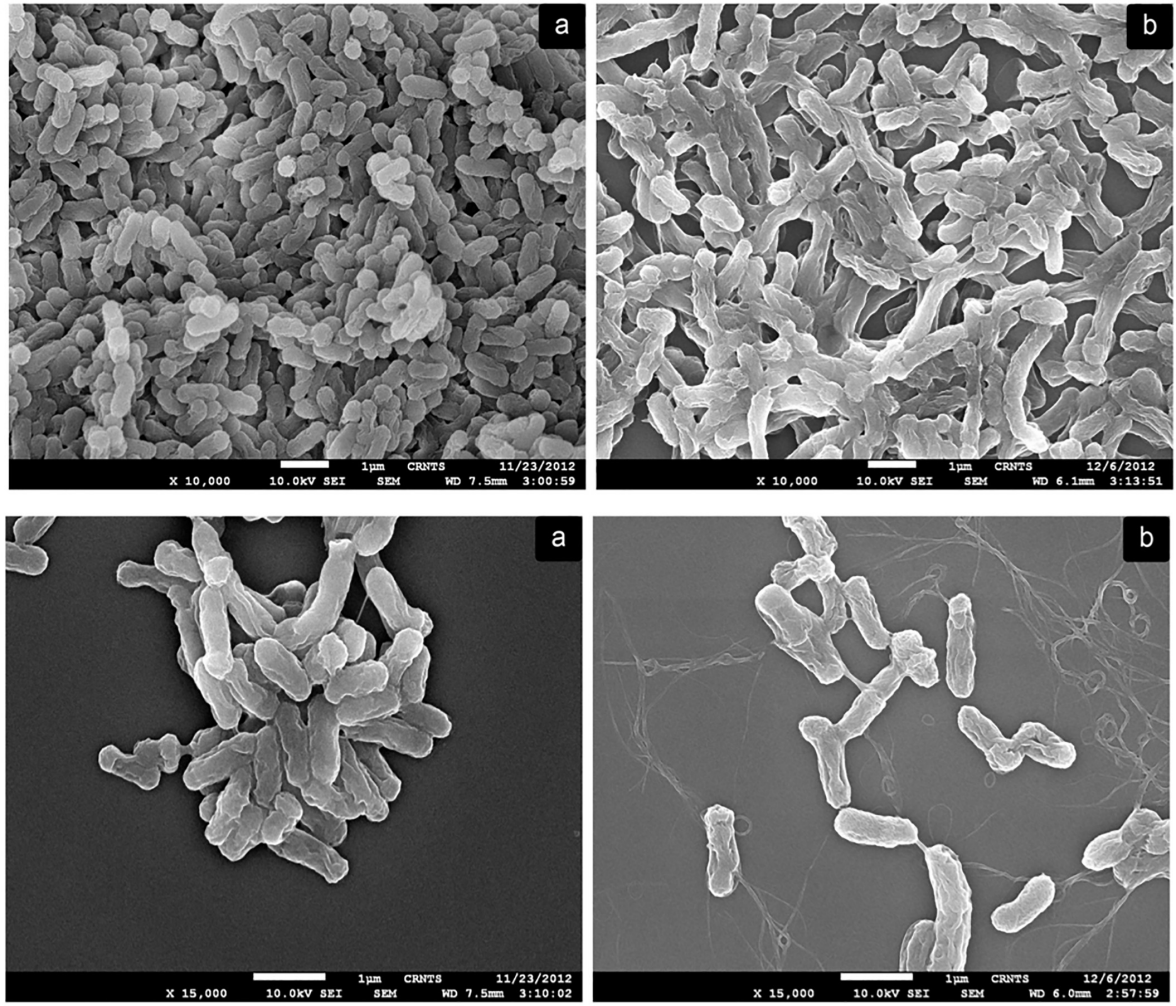
Scanning electron micrographs of isolate SAR1 before and after treatment with Cr. a: Control; b: After treatment with Cr.

The cell wall of bacteria represents the first defense system against any external stress [25]. The capsules or slime layer produced by some bacteria around the cell wall act as important metal binding sites. These substances also termed as slime layer, glycocalyx or extracellular polysaccharide (EPS), are mainly composed of polysaccharides [26]. SEM technique was employed to observe the changes in cell morphology of rhizobia in the presence of metals. EDS was carried out to check for the the presence of bound Cr. The SEM micrographs revealed morphological changes on exposure to Cr. Batool et al. observed *Ochrobactrum intermedium* Rb2 cells to increase in length with projections on cell surface along with increased production of capsular material which increased the cell wall thickness [27]. In our study also, the cells of isolate SAR1, on exposure to Cr, showed a large amount of production of capsular material leading to irregular morphology and aggregation of two cells. An increase in the size of the cell was also observed after metal exposure (Fig 1). This could probably be due to the excess polysaccharides that were seen surrounding the cells [27]. Similar morphological changes like increase in size and secretion of EPS was also observed in *Sinorhizobium* sp. BEL5B in presence of Ni stress [17]. Chen et al. have reported that the deformation of the cells in the presence of metals could be because of these secretions [28]. In an earlier study, a similar observation by Helmann et al. was reported for the production of these exopolysaccharides as cell’s strategy to survive the metal stress conditions and continue to perform their normal metabolic activities [29]. The EDS data did not show any peaks of Cr. This probably indicates the absence of adsorbed Cr ions on the biomass. This finding is in contrast to that obtained for *Sinorhizobium* sp. BEL5B for nickel biosorption, where Ni ions were biosorbed on the surface of biomass [17]. This probably could be because of biotransformation of adsorbed Cr (VI) to Cr (III), and release of Cr (III) into the solution which lead to its absence on the biomass [30]. This mechanism can be further confirmed by total Cr analysis and kinetic studies as described later.

### Chromium removal studies

#### Effect of pH

**Fig 2:**
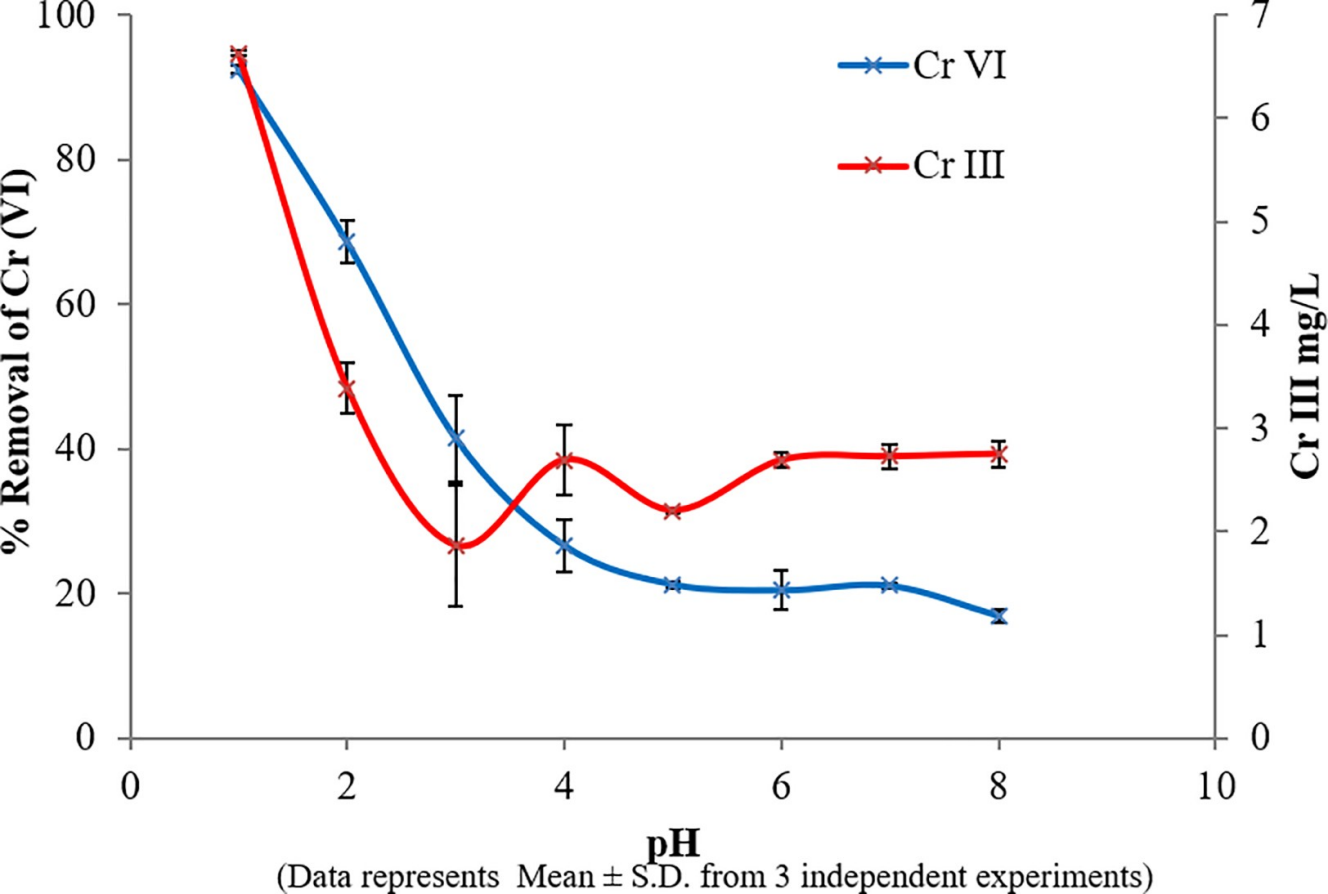
Effect of pH on bioremoval of Cr SAR1.

The metal ion solubility and surface charge of the biomass are significantly affected by the pH of the solution [31]. In our study, it was observed that Cr (VI) was more favoured at acidic pH 1 (Fig 2) where the maximum Cr (VI) reduction of 92% occurred. It was reduced to 17% at pH 8. A previous study has reported the existence of Cr (VI) as CrO_4_^2-^, HCrO_4_^-^, H_2_CrO_4_^2-^ and Cr_2_O_7_^2-^ in solution. Among these, HCrO_4_^-^ is the most prevalent form which shifts to CrO_4_^2-^ and Cr_2_O_7_^2-^ with increasing pH [32]. The initial mechanism of Cr (VI) removal involves the biosorption of Cr (VI) to the biomass and thereafter a reduction to Cr (III) [33]. At lower pH, there is increased protonation of the carboxyl and amino groups on the cell wall of the biomass resulting in the electrostatic attraction between the anionic Cr (VI) and the biomass, leading to an increased biosorption and hence increased bioreduction [34]. But as the pH increases, there was an increase in the negative charge of the biomass leading to repulsion between the negatively charged Cr (VI) and the biomass, causing a decrease in biosorption. A similar phenomenon was observed in this study as well, whereby an increase in pH caused a decrease in Cr (VI) removal. Hence, for further studies, a pH of 1 was used.

#### Effect of temperature

The study of the effect of temperature on biosorption did not show any significant changes in biosorption of Cr for the temperature range selected. Other authors have stated that temperature generally does not influence biosorption between the range of 20°C and 35°C^.^ Hence, on the basis of these results, further studies were carried out at 25°C ± 2°C [35, 36]^.^

#### Effect of biomass dosage

**Fig 3:**
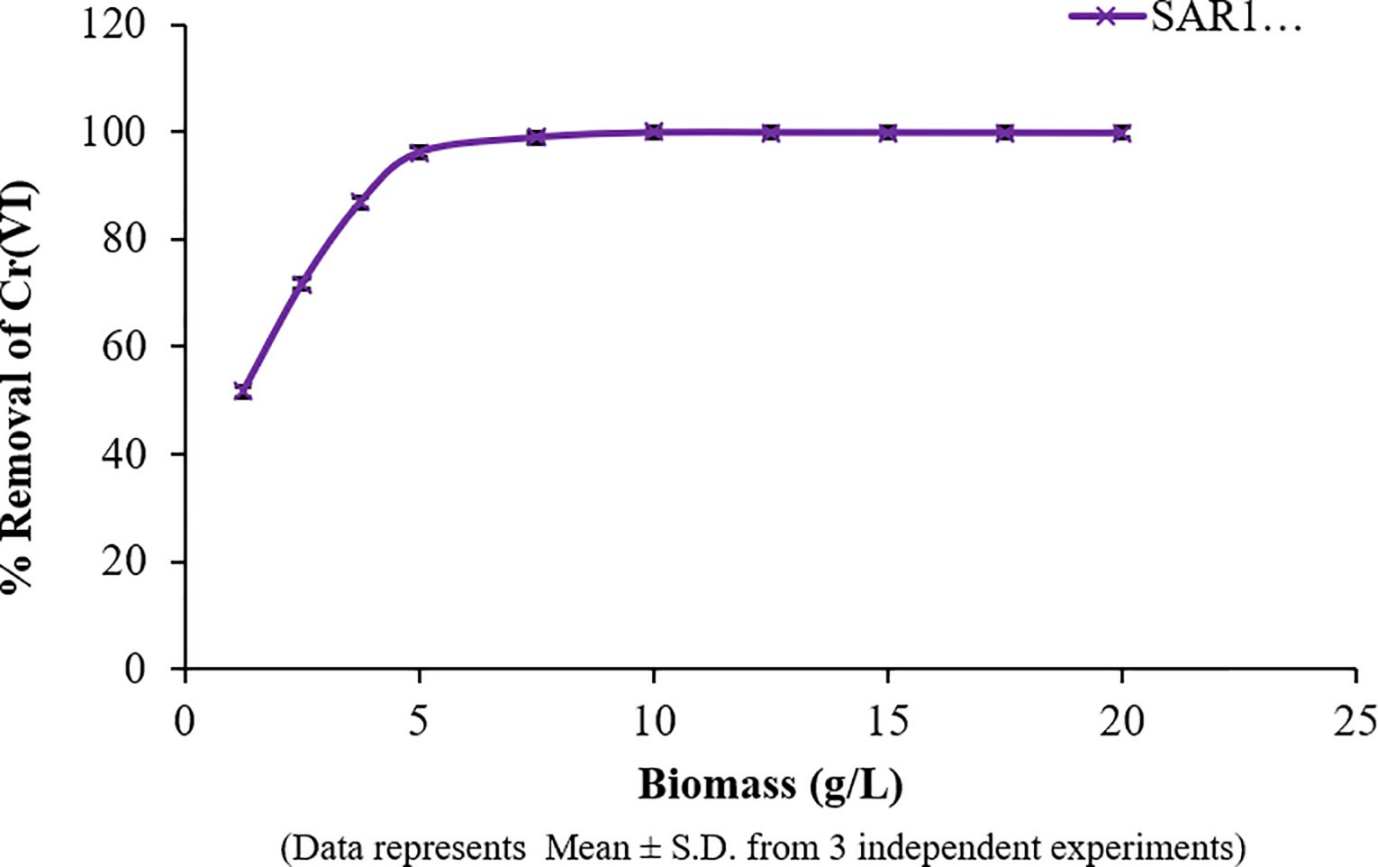
Effect of biomass dosage on bioremoval of Cr.

The third most important parameter that was studied for efficient biosorption was the biomass dosage. It was observed that biomass dosage and biosorption were directly proportional to each other. An increase in biomass dosage from 1.25g/L to 20g/L led to an increase in Cr (VI) removal; however, at higher concentrations of biomass, the increase was not significant (Fig 3). Burno et al. affirmed the increasing number of available sites for adsorption of the metals [37]. Based on the results, a biomass concentration of 7.5g/L was found to be efficient for Cr (VI) removal and was chosen for further studies [38].

#### Effect of different concentration

**Fig 4:**
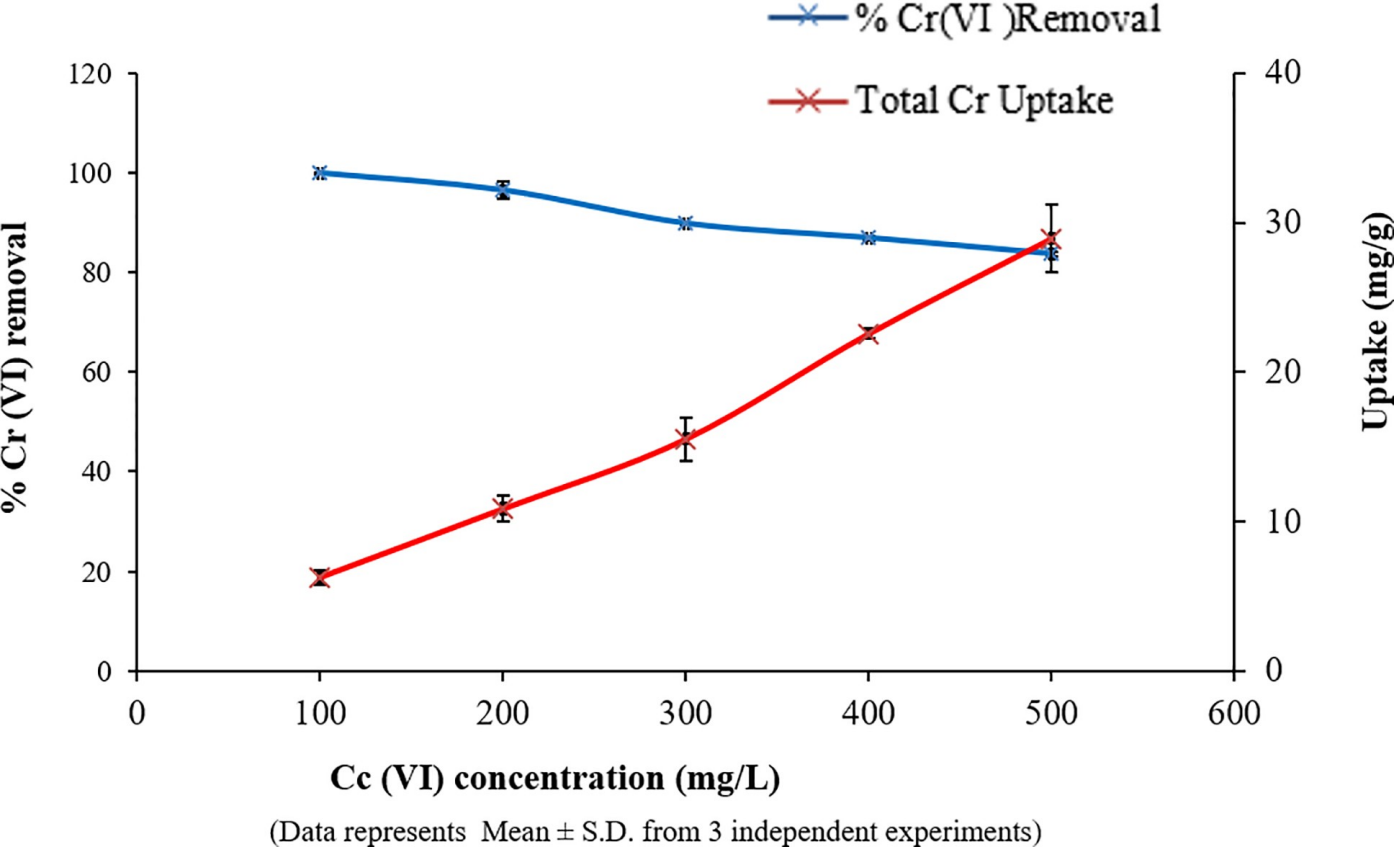
Effect of Cr concentration on bioreduction and uptake by SAR1.

Applying the above optimized conditions, biosorption study was carried out for different concentrations of metal. It was noted that an increase in metal concentration, from 100mg/L to 500mg/L, led to a decrease in % Cr (VI) removal (from 99.88% to 83.69%). But analysis of total Cr showed that uptake capacity of the biosorbent SAR1 increased (6.27mg/g to 28.95mg/g) with an increase in Cr (VI) concentration (Fig 4, S2 Table). These findings indicate a biosorption and bioreduction mechanism for Cr (VI) removal which is explained in the last section of this study, in detail, under the heading ‘Chromium removal by SAR1’. This trend was probably due to the saturation of adsorption sites at higher concentration of metals [39, 40]. It is reported that at low concentrations, biosorbent sites take up the available metal ions more quickly. However, at higher concentrations, metal ions need to diffuse to the biomass surface by intra-particle diffusion and greatly hydrolyzed ions diffuse at a slower rate leading to a decrease in percent biosorption [41].

#### Isotherm and kinetic studies for Cr removal

**Fig 5:**
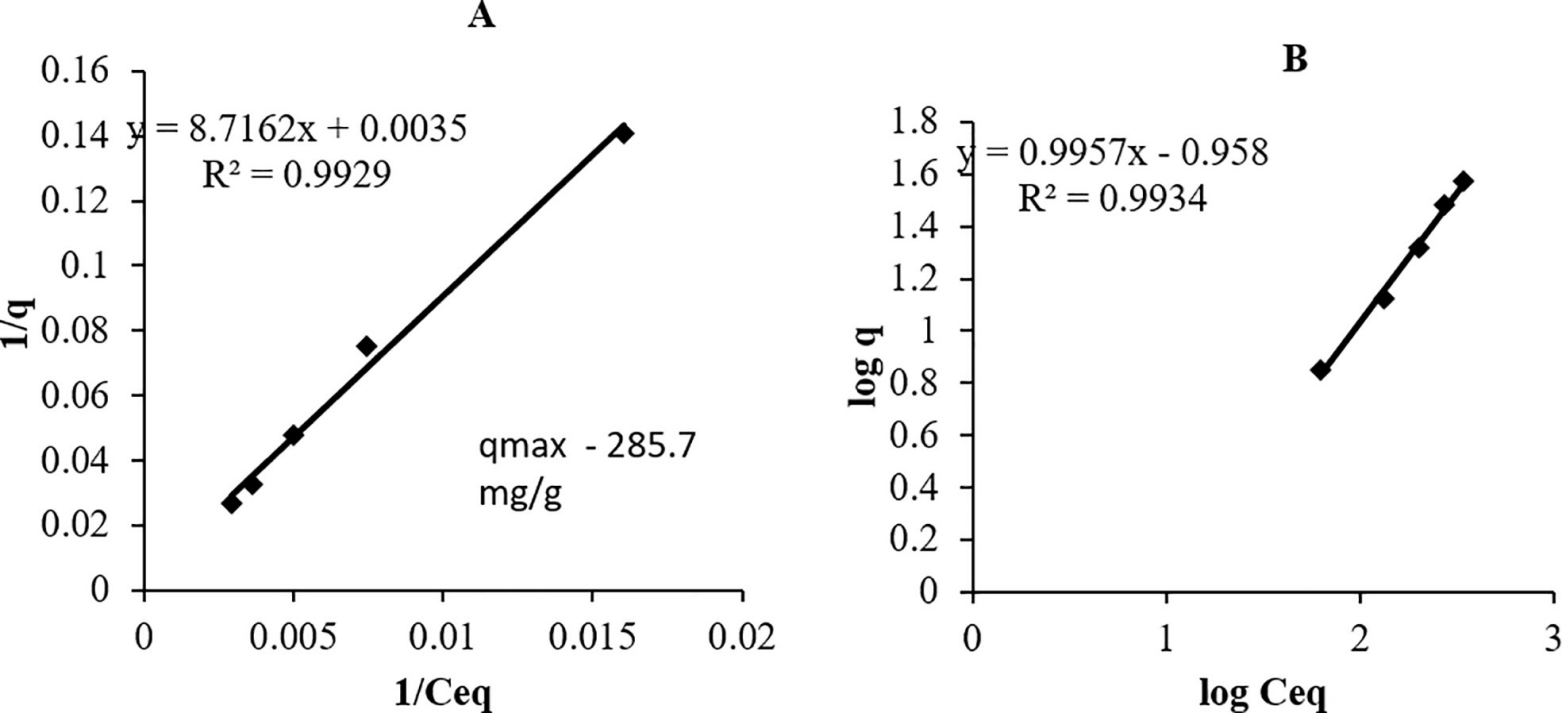
Adsorption isotherm for isolate SAR1 after Cr biosorption A: Langmuir isotherm; B: Freundlich isotherm.

The experimental data for biosorption of Cr by *Sinorhizobium* sp. SAR1, showed a R^2^ value to be high in case of both the isotherm models. For Freundlich isotherm the line crossed below the origin giving a negative y-intercept (Fig 5). Hence, the constant K value which measures the strength of biosorption obtained for Freundlich was found to be very low, indicating minimal adsorption and probably signifying only monolayer biosorption Hence the Langmuir model was found to best fit the experimental data. This also indicates that the biosorption of Cr (VI) onto SAR1 was by chemisorption, as chemical adsorption involves monolayer coverage [20]. Also, the 1/n value was found to be 0.9957, i.e. almost equal to 1, indicating that percent biosorption for total Cr remains constant and does not decrease with an increase in metal ion concentration (S4 Table). This is the first report of Cr removal by a *Sinorhizobium* sp. The biosorption capacities obtained in earlier reports were 284.4mg/g for *Aeromonas caviae* and 143mg/g for *Staphylococcus xylosus* [42, 43]. The q_max_ obtained in this study was found to be 285.71mg/g.

**Fig 6:**
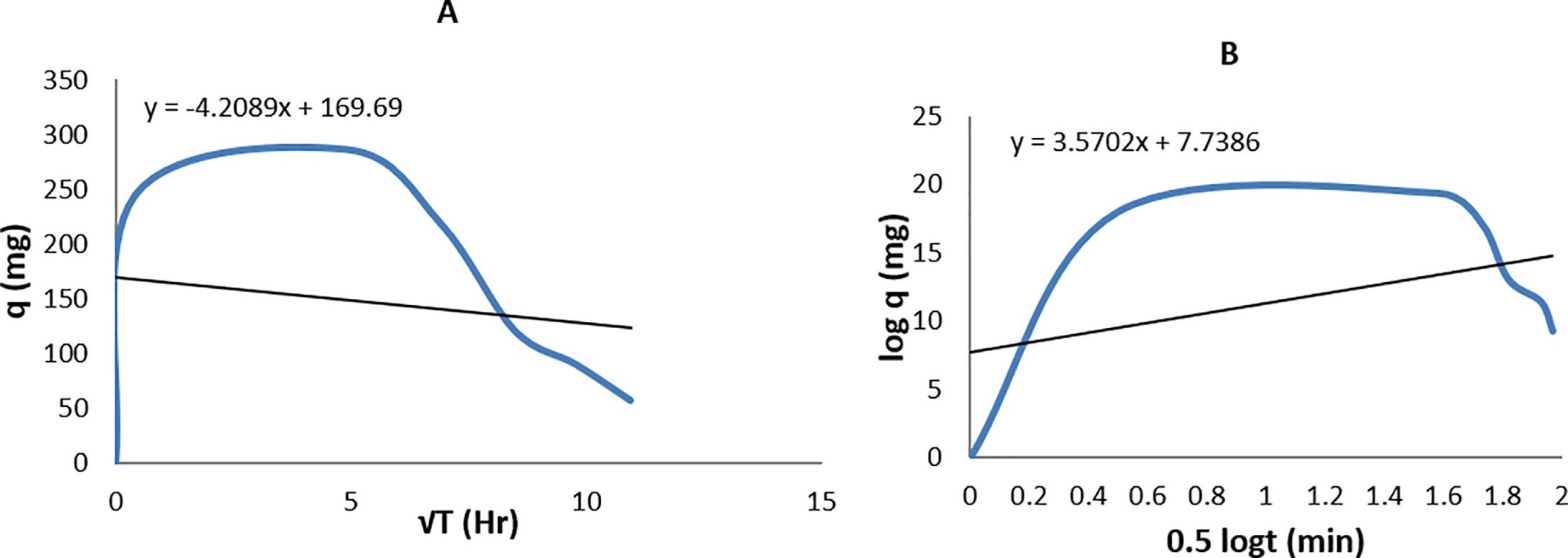
a. The plot of Q vs √t; Weber and Morris for the rate-limiting step; b. The plot of log Q vs 0.5 log t; Weber and Morris for the mechanism of adsorption.

The prediction of the rate-limiting step is an important factor in the adsorption process. The adsorption phenomenon of solid-liquid process with metallic ions as solutes is governed by the film diffusion or intra-particle diffusion (Godea and Pehlivan 2006) [44]. The most commonly used technique for identifying the mechanism involved in the adsorption process is by Webber and Morris plot [45]. According to this, if the rate-limiting step is intra-particular diffusion, then Fig 6A will be linear with the line passing through the origin. Otherwise, if Fig 6A shows an intercept, then the intra-particle diffusion, as well as other kinetic models, may be controlling the rate of adsorption, and all of these may be operating simultaneously.

In many processes the adsorption phenomenon may follow initially as intra-particle diffusion (pore diffusion) and later on film diffusion as a main governing phenomenon, as observed in the current study with Cr as a solute; indicated in Weber and Morris Fig 6B. The dual mechanism of Cr (VI) adsorption was also followed by desorption phenomenon; as there was a drop in the Q value observed in Weber and Morris Fig 6A & 6B. The phenomenon of desorption may be due to the ionic repulsion of the Cr ions which is observed after 5hr. Singh et al. also found both film and pore diffusion to be involved in the removal of arsenic (V) from aqueous solutions by haematite and feldspar [46].

The main phenomenon of the adsorption for Cr is the film diffusion, which signifies that a chromatographic process can be developed by using a flow rate with high throughput so as to achieve the maximum adsorption of Cr by increasing mass transfer and reduced desorption.

### FTIR analysis

Based on the literature cited, the bacterial cell wall components were found to aid biosorption. The peptidoglycan, teichoic acid, polysaccharides, lipopolysaccharides etc. have been found to be the major contributors for metal binding. The different anionic ligands such as OH^-^, CO_3_^2-^, HCO_3_^-^, PO_4_^3-^, SO_4_^2—^ and S^2-^ have been found to bind metals [47]. The vibrational frequency changes obtained in the functional groups of the biosorbents *Sinorhizobium* sp. SAR1 Cr- bound and Cr- free biomass is shown in Fig 6. For Cr removal by SAR1, along with the hydroxyl and the amino group (3431.73cm^-1^), the carboxyl (1302.7cm^-1^), the phosphate (1032.6cm^-1^) as well as the aromatic groups (860–680 cm^-1^) were found to contribute to metal binding. Previous studies have also emphasized on the involvement of phosphate groups in metal binding in gram-negative bacteria [12, 48]. Park et al. reported the role of carboxyl and amino groups on the cell surface in binding Cr (VI) [34]. As seen earlier, Cr (VI) removal is optimum at pH 1. These groups become protonated at low pH and thus aid in binding of Cr (VI) anions. Zang et al. reported complexation of carboxyl groups with Cr (VI) as a precondition for Cr (VI) reduction [49].

**Fig 7:**
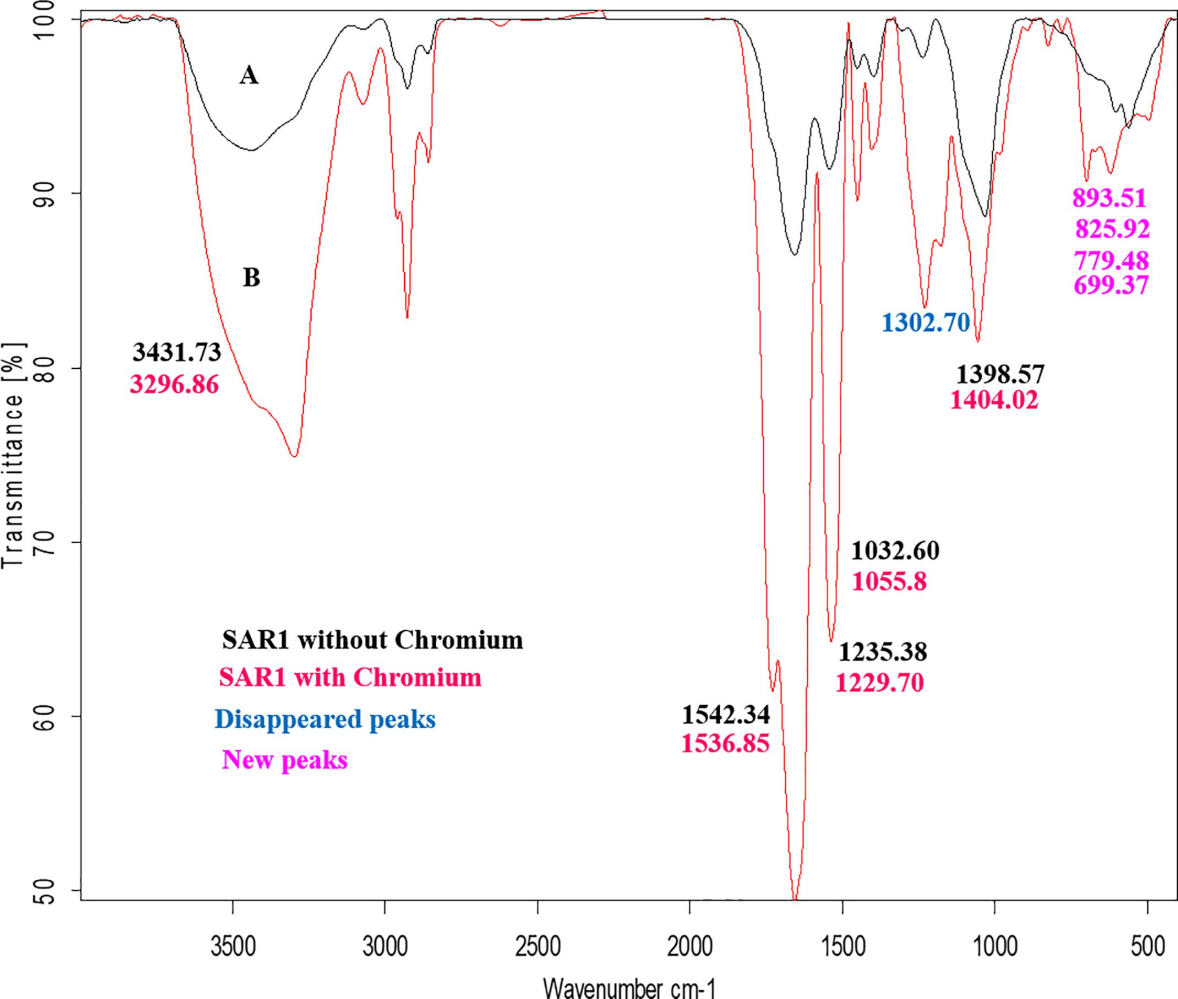
FTIR Spectra of isolate SAR1 A: before Control (–) and B: after Cr biosorption (–).

The other change noted was the weakening of peak observed at 1398 cm^−1^ (Fig 7). An earlier study by Han et al. has attributed this peak to carboxylated functional groups which sequester the Cr (III) formed from bioreduction of Cr (VI). Even in our present investigation, similar complexation of the carboxylate groups by Cr (III) seems to be a possibility. The changes in absorption frequencies obtained for Cr and their corresponding functional groups as well as the major components of the cell bearing these functional groups are summarized in Table 1. Another characteristic noted for all the three metals was the lowering of %transmittance of peaks after treatment of the biomass with metal, which is seen as a loading effect of the metals [50]. The major locations of these functional groups were found to be on the lipopolysaccharide and exopolysaccharide layers of both the isolates.

**Table 1:** Change of IR absorption bands after treatment of isolate SAR1 with chromium.

Original wave No. (cm^-1^)	Changed wave No. (cm^-1^)	Bond	Functional group	Class of compounds
3431.73	3296.86	N-H, O-H	Amine, Hydroxyl	LPS, peptidoglycan
1542.34	1536.85	N-H	Amine	Lipoprotein, peptidoglycan
1302.7	—————	C-O	Carboxyl	LPS, lipoprotein,
1235.38	1229.7	C-O	Ether (aromatic)	LPS, peptidoglycan
1032.6	1055.8	P-O	Organic phosphate	Lipoprotein (phospholipid)
	893.51, 825.92, 779.48, 699.37	C-H	Aromatic	Exopolysaccarides, LPS, lipoprotein

#### Chromium removal by SAR1: A dual mechanism of adsorption and bioreduction

**Fig 8:**
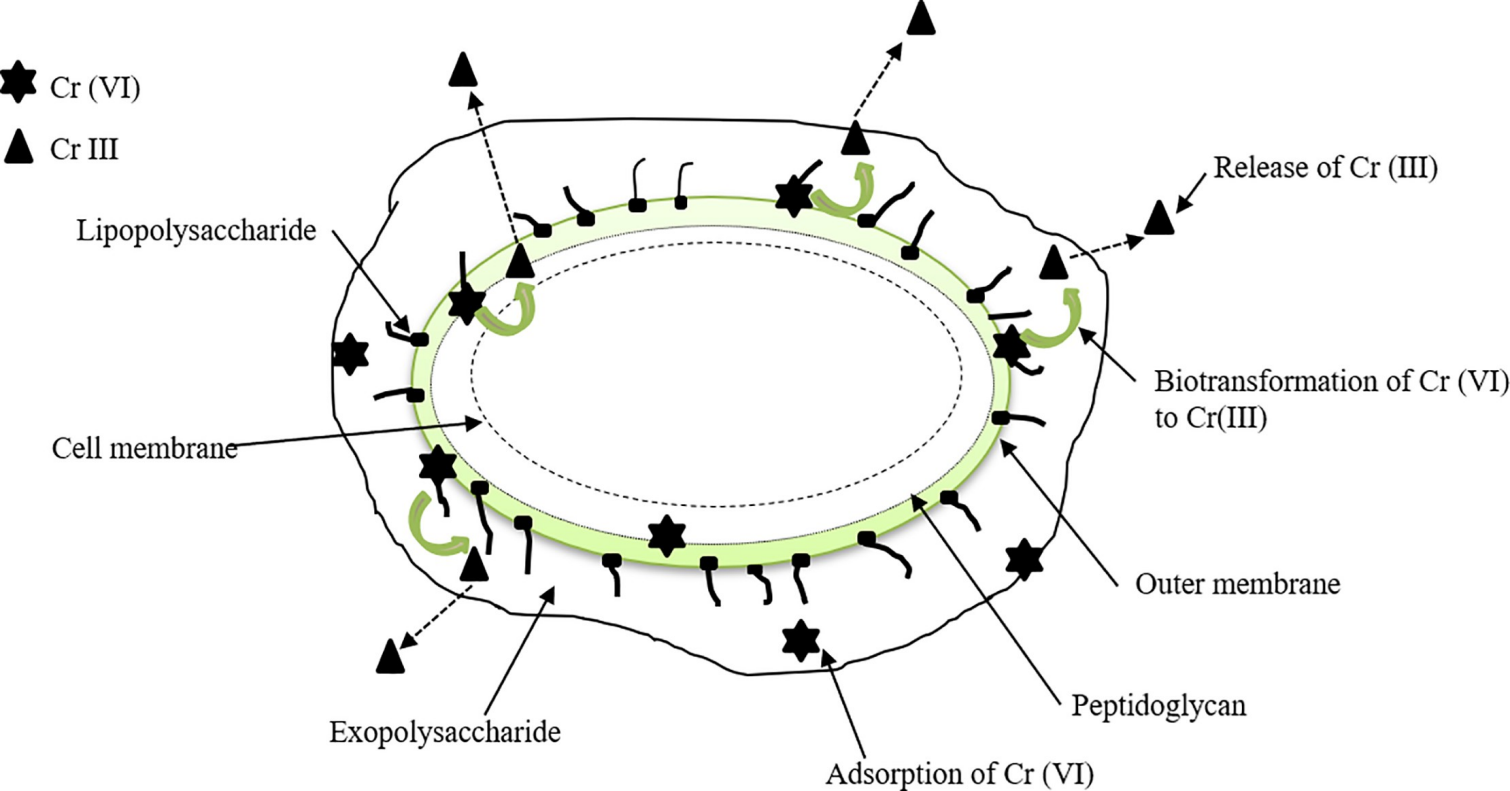
Steps in chromium biotransformation mechanism. a. Adsorption of Cr (VI) to exopolysaccharide, lipopolysaccharide, lipoproteins, peptidoglycans; b. Biotransformation of Cr (VI) to Cr (III); c. Release of Cr III into the solution.

On the basis of the results obtained, a probable mechanism of Cr (VI) removal in *Sinorhizobium* sp. SAR1 is depicted in Fig 8 [7]. The role of cell wall components of *Sinorhizobium* sp. SARI viz. exopolysaccharides, lipopolysaccharides and peptidoglycan, in adsorption of Cr (VI), is highlighted by the results of FTIR. The isotherm studies proved the adsorption of Cr (VI) onto cell wall components to be monolayer. Cr is known to exist in various oxidation states ranging from Cr (II) to Cr (VI). Among these states, Cr (III) and Cr (VI) are the most common and highly stable. The experimental data for Cr removal studies was initially estimated for Cr (VI) by the DPC method. DPC is a very sensitive method which detects only Cr (VI) and does not react with any other forms of Cr. An ICP-AES analysis was also done for the same solutions which estimated total Cr (all forms of Cr). Experiments done, showed a decrease in Cr (VI) in the solution after exposure to *Sinorhizobium* sp SAR1 biomass. This decrease can occur due to two reasons; adsorption of Cr (VI) to cell wall components and biotransformation of Cr (VI) to Cr (III). If only adsorption was occurring then the absence of Cr (VI) after DCP analysis should correspond with the same absence in ICP-AES analysis, but this was not the case. Instead, ICP-AES showed a higher amount of Cr, thus indicating the presence of other forms of Cr mostly Cr (III) since that is the most stable. This can only happen when biotransformation of Cr (VI) occurs, thus proving the dual mechanism of adsorption and bioreduction.

Another important observation made during the study was that the amount of Cr (VI) reduced was more than the amount of Cr (III) detected for all the concentrations studied. For example, in case of 100mg/L of initial Cr (VI), a 99.88% bioreduction of Cr (VI) was observed, but only 65.88 mg/L of Cr (III) was detected. This probably indicates that not all the adsorbed Cr (VI) was reduced to Cr (III) and some of the reduced Cr (III) after biosorption was released into the liquid solution. The desorption of Cr (III) by the isolate is further reinforced by a decrease in Q value of Weber and Moris plot. A similar observation is reported by Han et al. in which biotransformation of Cr (VI) by microalgae *Chlorella miniata* is explained [33]. Based on these results, it can be concluded that Cr (VI) removal by *Sinorhizobium* sp. SAR1 is a process involving biosorption as well as bioreduction or biotransformation. The sequence of removal might be in the order viz. (i) biosorption of Cr (VI) at low pH on the protonated sites of cell wall components of biomass; (ii) bioreduction of Cr (VI) to different intermediates and finally a stable form Cr (III) which probably occurs by the reductants on the biomass and enzymes in the periplasm, and finally; (iii) release of some of the Cr (III) into solution.

## Conclusion

The *Sinorhizobium* SAR1 sp. was found to be resistant to hexavalent Cr. The SEM analysis done, in the present study, suggests a physiological adaptation of the isolate to Cr stress. The use of such metal resistant bacteria not only offers genetic attributes for metal removal but can also withstand and multiply in the presence of heavy metals. The isolate was able to reduce hexavalent Cr to a less toxic trivalent form. The mechanism of bioremediation of Cr, by *Sinorhizobium* SAR1 sp., was found to be biosorption to various cell wall components followed by biotransformation of Cr (VI) to Cr (III), and finally the release of Cr III. These studies can help design an efficient Cr removal system using root nodule bacteria and be helpful for further investigations using industrial effluents. Knowledge of the same can hence make this system commercially exploitable.

## Supporting information

S1 Table Plate MIC values for 22 isolates. (PDF)

S2 Table Effect of Cr concentration on bioreduction and uptake by SAR1. (PDF)

S3 Table Adsorption isotherm for isolate SAR1 after Cr biosorption A: Langmuir isotherm; B: Freundlich isotherm.
(PDF)

S4 Table Adsorption constants estimated from the Langmuir and Freundlich equation for Biosorption of Cr by SAR1. (PDF)

S1 Fig Growth curve of SAR1 under Cr stress. (TIF)
