# Supplementary material for: Biotransformation of chromium by root nodule bacteria Sinorhizobium sp. SAR1
Source: PLoS One. 2019 Jul 30;14(7):e0219387. doi: 10.1371/journal.pone.0219387 (PMC6667149; doi:10.1371/journal.pone.0219387)
Supplement: S1 Table — (PDF) [file pone.0219387.s001.pdf]

S1 Table. Plate MIC values for 22 isolates

Isolates	Cr concentration mM
**KHA1**	0.5
**KHA2**	0.5
**KAL1**	0.5
**TUR1**	0.5
**TUR2**	0.5
**CRO1**	0.5
**CRO2**	0.5
**UNQ3**	0.5
**SHE2**	0.5
**MUM1**	0.5
**SON2**	0.5
**BEL4**	1(+)
**BEL5B**	0.5
**LIN1**	0.5 (+)
**KAL2**	0.5
**KAL3**	1
**KAL5**	0.5
**MUM3**	1
**TAL1**	0.5
**SAR1**	1
**HIN2**	0.5
**KAL4**	0.5
