# Supplementary material for: Biotransformation of chromium by root nodule bacteria Sinorhizobium sp. SAR1
Source: PLoS One. 2019 Jul 30;14(7):e0219387. doi: 10.1371/journal.pone.0219387 (PMC6667149; doi:10.1371/journal.pone.0219387)
Supplement: S2 Table — (PDF) [file pone.0219387.s002.pdf]

S2 Table. Effect of Cr concentration on bioreduction and uptake by SAR1

Cr (VI)	% Cr (VI) Removal	SD	Uptake(mg/g)	SD
**100**	99.87786	0.480833	6.273333	0.480833
**200**	96.45694	0.895669	10.86	0.895669
**300**	89.78514	1.439669	15.522	1.439669
**400**	86.88574	0.303585	22.57467	0.303585
**500**	83.68899	2.234457	28.94667	2.234457
