# Supplementary material for: Biotransformation of chromium by root nodule bacteria Sinorhizobium sp. SAR1
Source: PLoS One. 2019 Jul 30;14(7):e0219387. doi: 10.1371/journal.pone.0219387 (PMC6667149; doi:10.1371/journal.pone.0219387)
Supplement: S3 Table — Adsorption isotherm for isolate SAR1 after Cr biosorption A: Langmuir isotherm; B: Freundlich isotherm. (PDF) [file pone.0219387.s003.pdf]

**S3 Table. Adsorption isotherm for isolate SAR1 after Cr biosorption A: Langmuir isotherm; B: Freundlich isotherm**

| Cr (VI) | 1/ceq | 1/q | logCeq | logq |
|---|---|---|---|---|
| **100** | 0.016028 | 0.140951 | 1.795115 | 0.850932 |
| **200** | 0.007457 | 0.07515 | 2.127429 | 1.124069 |
| **300** | 0.005003 | 0.047655 | 2.300791 | 1.321888 |
| **400** | 0.003653 | 0.032808 | 2.437307 | 1.484015 |
| **500** | 0.002928 | 0.026582 | 2.533454 | 1.575419 |
