# Supplementary material for: Biotransformation of chromium by root nodule bacteria Sinorhizobium sp. SAR1
Source: PLoS One. 2019 Jul 30;14(7):e0219387. doi: 10.1371/journal.pone.0219387 (PMC6667149; doi:10.1371/journal.pone.0219387)
Supplement: S4 Table — (PDF) [file pone.0219387.s004.pdf]

S4 Table. Adsorption constants estimated from the Langmuir and Freundlich equation for Biosorption of Cr by SAR1

**Langmuir**			**Freundlich**		
**q_{max} (mg/g)**	**b**	**R^2**	**K**	**1/n**	**R^2**
285.71	0.000402	0.9929	0.11	0.9957	0.9934
