# Supplementary figures and images for: Biotransformation of chromium by root nodule bacteria Sinorhizobium sp. SAR1
Source: PLoS One. 2019 Jul 30;14(7):e0219387. doi: 10.1371/journal.pone.0219387 (PMC6667149; doi:10.1371/journal.pone.0219387)

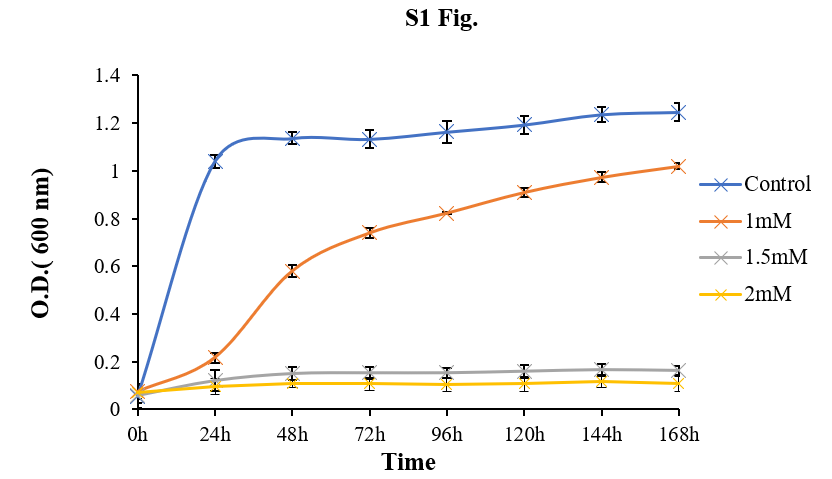

Supplement: S1 Fig — (TIF) [file pone.0219387.s005.tif]
